# Multidimensional Signal Processing and Applications

**DOI:** 10.1155/2014/365126

**Published:** 2014-04-30

**Authors:** Salah Bourennane, Julien Marot, Caroline Fossati, Ahmed Bouridane, Klaus Spinnler

**Affiliations:** ^1^Ecole Centrale Marseille, Université Aix-Marseille, Institut Fresnel-CNRS UMR 7249, Avenue Escadrille Normandie Niemen, 13013 Marseille, France; ^2^Department of Computer Science and Digital Technologies, Northumbria University, Pandon Building, Newcastle upon Tyne NE2 1XE, UK; ^3^Fraunhofer-Entwicklungszentrum Röntgentechnik EZRT, Fraunhofer-Instituts IIS, Flugplatzstr 75, 90768 Fürth, Germany

In our daily lives and almost unconsciously, we deal with multidimensional data. From color images converted to the luminance and chrominance format to magnetic resonance images commonly acquired for health purposes, from different fashions to write an alphabet to array processing signals underlying any telecommunication system, we deal with multidimensional data.

In this special issue, we tried to show the variety of the topics which are currently investigated with multidimensional signal processing tools. The mathematical tools presented in this issue are as diverse as adaptive detectors, wavelet processing, principal component analysis, and improved classical image processing tools such as histogram equalization.

In the array processing paradigm, a two-dimensional matrix containing the data depends on the polarization properties of the sources, their number, and the number of sensors in the receiving antenna. Hence the interest of a multidimensional representation, including a polarization variable with two or three possible values, and a real and a complex part for the source amplitudes.

In the image processing paradigm, data are as various as magnetic resonance or color images, whose representation can be transferred from the RGB (red green blue) format to other spaces emphasizing for instance the luminance or the chrominance. It is shown how magnetic resonance brain images are classified with support vector machine. To avoid problems related to high dimensionality, which is current in big data processing, adequate features are extracted from the data by discrete wavelet transform and principal component analysis. Color spaces, which are useful for skin detection, for instance, are also further investigated: whatever the representation space is, a color image is a third order tensor, in other words, a three-dimensional data. It is shown how to detect image splicing with the help of merged features in the chrominance space: the relationships between pixels in a neighborhood are studied with a Markov process and the extraction of DCT features from the chrominance channel. Then, with the help of new color spaces, it is shown how evolved versions of neural networks called extreme learning machines can fuse multiple information such as color and local spatial information from face images. The “multi” aspect can also appear in the image processing paradigm when multiple images are obtained from several parameters. In images provided by synthetic aperture radar exploited for flood detection, contrast enhancement is achieved by an adjustable histogram equalization technique. For such an application where the visual aspect of the results are much important much, a color image, that is, a multidimensional signal, can be built from several two-dimensional result images, to get an informative map, where the color informs on the nature of the imaged scene, flooded or not, for instance. Starting from images, a set of multidimensional data is extracted from Serbian texts: the Serbian alphabet, made of 30 letters, can be expressed in a Latin or in a Cyrillic fashion. All letters can be classified into four sets. By studying the frequencies of occurrence of each type of letter in a text, one can deduce that this text is written in the Latin or the Cyrillic fashion. In this application, matrices describing the cooccurrence in the distribution of the four types of letters are built out of any text, to make use of the classical texture features. Adapting the texture features to such a text recognition application, introducing a parameter which is the writing fashion, is a brand new idea. The “multi” aspect can also relate to multiresolution. Histogram of oriented gradients and hue descriptors can be merged to combine information related to the shape of an object and its color. By computing the merged data at several resolution levels, an innovative multidimensional descriptor is obtained. An application considered in this special issue is aircraft characterization and detection of images.

In a nutshell, the “multi” representation attracts the interest of researchers from very diverse application fields. Hopefully, this special issue will contribute in diffusing the models and tools of multidimensional signal processing to various application fields.

*Salah Bourennane* *Salah Bourennane* *Julien Marot* *Julien Marot* *Caroline Fossati* *Caroline Fossati* *Ahmed Bouridane* *Ahmed Bouridane* *Klaus Spinnler* *Klaus Spinnler*

